# Tackling NHS winter pressures due to respiratory disease: the UK Centre for Applied Respiratory Research Innovation and Impact (CARRii)

**DOI:** 10.1038/s41533-026-00500-x

**Published:** 2026-04-02

**Authors:** Paul Stephenson, Monica Fletcher, Stuart D. Faulkner, Chris Griffiths, Aziz Sheikh

**Affiliations:** 1 Nuffield Department of Primary Care Health Sciences, University of Oxford, Oxford, UK; 2 The UK Centre for Applied Respiratory Research, Innovation and Impact (CARRii), University of Oxford, Oxford, UK; 3 Primary Care Respiratory Society, London, UK

**Keywords:** Diseases, Health care

## Abstract

Seasonal respiratory viral infections are the major cause of increased pressure on national health systems such as the UK’s national health service (NHS) during winter months. Emergency departments and hospitals are full as increasing numbers of patients require hospitalisation for lower respiratory tract infections and exacerbations of COPD, asthma, and other respiratory conditions. These winter pressures are largely predictable. Forecasting models give healthcare managers the chance to mitigate winter pressures by allocating resources more effectively. Despite this predictability and the production of NHS resilience plans, the UK is particularly susceptible to winter pressures. Communities facing social and environmental disadvantage are at increased risk of hospitalisation. Despite the focus on real-life research and guideline dissemination and implementation over the last 20–30 years, care of patients with respiratory conditions in the UK remains sub-optimal. To tackle winter pressures, a different approach is needed. The UK Centre for Applied Respiratory Research Innovation and Impact (CARRii) is a new UK-wide network which aims to drive policy change and use innovative ways of implementing research to achieve maximum impact on patient care. Its missions are to reduce NHS winter pressures and respiratory health inequalities, and its focus is on three areas: prevention; self-management and connected care; and optimisation of clinical care. CARRii unites leading experts across respiratory research, clinical medicine, data science, public health, industry innovation, and patient advocacy. CARRii’s first Annual Scientific Meeting was held recently. Implementation of research is more likely to succeed if it is based on real-world data, there is multi-agency involvement in its design and implementation, it is patient-focussed, and policy makers are convinced of its benefits. By positioning implementation and impact as a central scientific goal, CARRii aims to show how healthcare systems can deliver respiratory solutions at scale. This requires investment and cross-sector collaboration. If successful, this approach will reduce winter pressures, improve health equity and strengthen system resilience.

## Introduction

The 2025/2026 winter season has been one of the most difficult winters to date experienced by the UK’s National Health Service (NHS)^1^. This article focusses on winter pressures caused by respiratory communicable diseases such as the recent influenza A subclade K variant. Despite our ability to predict these winter pressures, and the focus on real-life research and guideline implementation over the last 20–30 years, we highlight the fact that care of patients with respiratory conditions in the UK remains sub-optimal and that implementation of research which could improve health outcomes and reduce winter pressures is inadequate. The Centre for Applied Respiratory Research Innovation and Impact (CARRii) is a new UK-wide network which aims to drive policy change and use innovative ways of implementing research with maximum impact on patient care. Its missions are to reduce NHS winter pressures and the burden of unscheduled care in a way that reduces longstanding respiratory health inequalities. We summarise the rationale for CARRii, its themes and specialist platforms, present the conclusions following our first Annual Scientific Meeting held in October 2025, and highlight the challenges and our priorities for the future.

## The prevalence of respiratory diseases

The 2023 update of the Global Burden of Disease Study^2^ confirms that two of the top five leading causes of age-standardised death worldwide are respiratory conditions: chronic obstructive pulmonary disease (COPD) is ranked third, with an age-standardised mortality rate of 38.4 per 100,000 population [95% confidence intervals 33.2–45.8]; and lower respiratory tract infections (LRTIs) are fourth, (mortality rate 31.6 [28.3–35.3]). COVID-19, having been the leading cause of age-standardised death in 2021, is now ranked 20^th^. LRTIs cause 2.60 million deaths annually, with *Strep pneumoniae*, *Haemophilus influenzae*, and the influenza virus being the three leading pathogens^3^.

Asthma, COPD and interstitial lung disease (ILD) are the three most common chronic lung diseases worldwide^4^. In the UK, prevalence rates for asthma vary between 7.0% and 15.9%, for COPD between 3.0 and 5.1%, and for ILD between 0.3 and 0.6%^5^. Nevertheless, rhinitis is the most common chronic disease in the UK, with an estimated adult prevalence of 26%^6^, in line with worldwide prevalence ranges^7^. All these diseases affect different parts of the ‘unified airway’^8^, and rhinitis is a common co-morbidity of asthma (as per the Allergic Rhinitis and its Impact on Asthma (ARIA) guidelines^9^) and COPD^10^. In a recent study of over 2500 patients, 70% of asthma patients and 58% of COPD patients reported co-morbid rhinitis which increased their exacerbation risk significantly (OR [95% CI] 1.38 [1.02 – 1.86] and 1.57 [1.23–2.01], respectively)^11^.

## Winter viruses

Annual epidemics of the common cold, influenza and respiratory syncytial virus (RSV) affect populations every winter, with occasional pandemics of severe acute respiratory syndrome coronavirus (SARS-CoV) and more recently SARS-CoV-2 adding to the mix. The mechanisms underlying this seasonality have been reviewed recently^12^. Lower temperatures and humidity levels cause hypersecretion of mucin, impairment of mucociliary clearance, a reduction in host defence capability^13^, increased replication of rhinovirus^14^, and impairment of host immune response to influenza^13^. Winter viruses such as influenza and RSV are much more stable at low humidity levels compared to summer or all-year viruses such as adenovirus or enteroviruses^12^. There is a clear association between wintertime reduction in absolute and relative humidity and epidemics of influenza-related mortality^15^.

## Winter pressures

Acute respiratory viral infections such as influenza are the major cause of increased pressure on national health systems during winter months. They cause increasing numbers of LRTIs^16^ and exacerbations of COPD^17^, asthma^18^, and other respiratory conditions such as bronchiectasis^19^, leading to increased risk of hospitalisation and death. Younger children, older adults, those from deprived backgrounds, and patients with immunosuppression and multimorbidity are most at risk^20^. In England and Wales, the number of hospital admissions for COPD and asthma exacerbations combined increased by 82% between 1999 and 2020 – from 210,000 to 383,000 per year – and COPD patients with acute LRTI accounted for 38.7% of all hospital admissions^21^.

Non-respiratory factors are involved too. Norovirus is the commonest cause of viral gastroenteritis. Increased rates of norovirus infection, leading to hospital admission in the most severe cases, are associated with lower temperatures and lower humidity levels^22^ (like influenza) – hence its common name of ‘winter vomiting disease’. Lower temperatures also affect the incidence and outcomes of diseases such as myocardial infarction^23^, and the elderly, deprived and vulnerable are more at risk from strokes, hypothermia, and injuries due to falls^24^. NHS staff experience higher rates of illness during winter^25^, which (combined with pre-existing staff shortages) leads to reduced workforce capacity.

The NHS is particularly susceptible to winter pressures, with emergency departments crowded, corridors full^26^, and hospitals on full alert as each winter occurs. The UK has among the lowest number of hospital beds of any country in Europe (2.4 per 1000 population), on a par with northern European countries but lower than the European Union average (5.1/1000)^27^. This implies a hospital system with efficient use of fewer beds, but it does mean there is little spare capacity – and when hospitals have very few spare beds, patients with respiratory problems are more likely to be cared for on non-respiratory wards, have less access to respiratory specialists, and receive sub-optimal discharge planning^28^, thus making their care *less* efficient. Winter pressures arise not only from hospital admissions but also from each patient’s length of hospital stay. Older adults with COPD exacerbations and LRTIs – the main patient group adding to winter pressures^21^ – have much longer hospital stays than younger patients^29^. A significant proportion of these patients will require additional social care on discharge, and delays in arranging this due to lack of provision quickly lead to ‘bed blocking’ and further reductions in hospital capacity^30^.

## Predicting winter pressures

Therefore, winter pressures on the NHS, largely due to respiratory viral infections, are to be expected. Knowing the extent of these pressures – particularly the number and type of patients likely to require hospital admission – is essential for healthcare policymakers and system managers if they are to allocate resources more effectively and have appropriately trained staff to cope with them.

In 2008, a case-control study of older patients consulting medical services with a wintertime LRTI or exacerbation of chronic respiratory disease reported that 157 were hospitalised versus 639 controls who were not. The most important risk factor for admission was having COPD (OR 4.0 [95% CI 1.4–11.4]), other chronic disease (OR 2.9 [1.2–7.0]), or both (OR 6.7 [2.4–18.4]). Having two or more hospital admissions in the previous year and being housebound were also independent risk factors (OR 4.6 [1.3–16.0] and 2.2 [1.0–4.8], respectively)^31^.

The Early Pandemic Evaluation and Enhanced Surveillance of COVID-19 (EAVE II) Scottish national surveillance platform was used to predict those people at risk of hospitalisation and death during the COVID-19 pandemic^32^. The data platform was repurposed for a national retrospective cohort study to identify risk factors for hospitalisation due to acute respiratory infection during the winter of 2022/2023^20^. The factors identified – the young, elderly, those patients with multimorbidity and previous emergency admissions, and people living in areas of deprivation – are highly likely to be generalisable given the large dataset used.

The UK Health Security Agency developed a number of forecasting models to predict hospital admissions for COVID-19, influenza and RSV infection, and bed occupancy, two weeks ahead, in England over the 2023/2024 winter^33^. Admission forecasts, especially for RSV and influenza, were reliable, particularly at a regional level, and were communicated throughout the NHS and to the UK government.

Data from the southern hemisphere can be used to forecast winter pressures in the northern hemisphere six months later and can indicate the likely influenza strain involved. Having shown a rapid increase in incidence during the southern hemisphere 2025 influenza season, the influenza A(H3N2) subclade K variant dominated the 2025/2026 winter season in the UK^34^. Early post-vaccine effectiveness against influenza hospital admissions currently remains within typical ranges (72–75% in children and 32–39% in adults)^34^.

In March 2025, NHS England announced the roll-out of a new artificial intelligence (AI) software tool to be used during home care visits to predict patients’ risk of falls. The aim was to ‘achieve 97% accuracy’ and to ‘prevent up to 2000 falls and hospital admissions each day’^35^. However, the roll-out’s feasibility met with considerable scepticism^36^, highlighting the dangers of overestimating the value of predictive tools and underestimating the difficulties involved in their implementation.

The predictability of winter pressures provides an opportunity for healthcare policymakers to produce resilience plans^37,38^, including measures to prevent transmission of infection such as hand washing, social distancing and the use of face masks^39^ – whilst being aware of the potential problems with compulsory mask-wearing for those with physical or learning disabilities^40^.

## Health inequalities

Health inequalities exist throughout the year, but colder temperatures and winter virus infection add to the pressures on communities facing social and environmental disadvantage^41^. Cold homes with poor indoor air quality and exposure to damp, mould, pests or pollutants, significantly impair child respiratory health^42^. Patients with asthma with lower socioeconomic status have substantially increased emergency department attendance and hospital admission rates^43^. A recent Asthma and Lung UK survey showed that acute COPD exacerbation rates were higher in smokers, in patients with lower incomes, and in patients living in poor housing conditions^44^. Following discharge from hospital, these patients are most likely to need social support and integration of social care and primary care services, especially if they are housebound^31^.

Although primary care might have the potential to reduce health inequalities, existing evidence is scarce. A recent review^45^ suggested that reducing health inequalities requires five key principles: primary care services to be coordinated across the system; differences within patient groups to be recognised; making allowance for different patient needs; integrating patients’ views and cultural references; and engaging communities in service design and delivery.

## The challenge of optimising clinical care: translating research into practice

Translation of research findings into clinical practice, particularly in primary care, has improved markedly over the last 30 years. Developing consensus on national respiratory research priorities^46^, and establishing professional organisations which support and disseminate primary care-focussed respiratory research has been essential^47,48^. In 2000, the UK’s Primary Care Respiratory Society (PCRS) changed the name of its peer-reviewed academic journal to the *Primary Care Respiratory Journal*^48^. With the full support of the newly-formed International Primary Care Respiratory Group (IPCRG), a further name change in 2014, and transfer of ownership to the IPCRG in 2024, this Journal is now the only fully-indexed journal in the world dedicated to publishing research on the primary care management of respiratory diseases.

Nevertheless, implementing clinical guideline recommendations is a challenge. Some guidelines such as the Global Initiative for Asthma (GINA)^49^ and the Global Obstructive Lung Disease (GOLD)^50^ guidelines have received worldwide recognition. However, even by 2003, the dramatic increase in (largely secondary care-focussed) national and international guidelines led to various questions about their usefulness and relevance to primary care^51^. Consequently, the IPCRG produced guidelines for the diagnosis and management of chronic respiratory diseases in primary care in 2006^52^, with one paper focussed entirely on dissemination and implementation^53^. Since then the IPCRG has produced and disseminated guideline-aligned desktop helpers designed to meet the needs of primary care^54^, and this Journal has regularly published primary care-relevant guidelines to facilitate their implementation^55,56^. In the UK, the PCRS has a crucial role in educating primary and community healthcare professionals by providing various resources including clinical tools, practical guidelines, webinars and podcasts^57,58^.

## The importance of real-world evidence

Although well-executed randomised controlled trials (RCTs) are considered the cornerstone of evidence-based medicine (EBM)^59^, the importance of good quality real-life studies with wider inclusion criteria and findings relevant to general practice populations is now widely recognised^60^. The Respiratory Effectiveness Group (REG) has expounded the importance of real-world evidence for nearly 20 years^61,62^ and its members’ publications have had considerable influence – not least a study published 15 years ago on leukotriene antagonists as controller medication in asthma^63^. The REG has recently published guidance on how to incorporate real-life evidence into EBM^64^.

## The need for change

Producing and disseminating real-life evidence is one thing; making actual improvements in the quality of care and embedding this across health systems is another. In the UK, various aspects of health care including prevention, diagnosis and delivery – all of which are relevant to tackling winter pressures – still require improvement. Examples are:

- **Vaccination uptake**. Uneven and non-optimal rates of vaccination uptake persist^65,66^, not least because of inconsistent public health messaging and uninformed criticism of vaccination which is amenable to behavioural intervention^67,68^.
- **Access to point-of-care tests**. Access to rapid point-of-care diagnostic tests (POCTs) such as C-reactive protein (CRP), which can guide antibiotic prescribing for COPD exacerbations^69^, is amongst the lowest in Europe despite advice on implementation strategies being available^70,71^.
- **Sub-optimal care of asthma and COPD**. The findings of the National Review of Asthma Deaths (NRAD), published in 2014^72^, resonate with those from previous Confidential Enquiries^73^. Personal asthma action plans (PAAPs) were provided to only 44 (23%) of the 195 people who died during the study period, and no asthma review had taken place in general practice in the year before death in 43% of cases. There was excessive prescribing of short-acting beta-agonist (SABA) reliever medication, under-prescribing of preventer medication, and evidence of inappropriate prescribing of long-acting beta-agonist (LABA) bronchodilators^72^. A subsequent parallel cohort study of asthma treatment in the UK and France confirmed that SABA overuse was common, particularly in the UK, and that inappropriate LABA prescribing was still present – although more common in France and less than seen previously^74^. Another study showed that 72% of UK primary care patients with potential severe asthma had not been referred and/or had not had specialist review in the previous year^75^. As regards COPD, a large primary care database study of over 24,000 patients^76^ showed that some received no treatment despite being symptomatic, and the majority received inhaled corticosteroids irrespective of their airflow obstruction or exacerbation history, contrary to GOLD recommendations. The National Respiratory Audit Programme^77^ only uses primary care data from Wales, and the last Welsh audit was performed in 2017. Five years later, primary care practices from England, Scotland, and Northern Ireland were significantly worse than Welsh practices at coding lung function parameters used in COPD diagnosis and at referring appropriate patients for pulmonary rehabilitation^78^.
- **Lack of implementation of existing evidence**. Pulmonary rehabilitation (PR) is one of the most effective of all interventions for people with COPD, and is strongly recommended in international guidelines^50^. Its clinically significant benefits on health-related quality of life, exercise capacity, dyspnoea, fatigue, and emotional function, are now so well established that any more RCTs comparing PR and conventional care in COPD are considered unjustifiable^79^. However, PR referral rates are low internationally^80^, and between 2004 and 2014 only 9% of eligible COPD patients in England were referred to PR from primary care^81^. Barriers to PR referral include limited awareness of the clinical benefits, little knowledge of local PR providers, consultation time restraints, and presumed low patient motivation^82^.
- **Adoption of different service models**. Service models such as Acute Respiratory Infection Hubs have been shown to improve access, reduce healthcare inequalities and improve the quality of care for patients^83,84^, but adoption remains inconsistent.

## A new UK national network: the Centre for Applied Respiratory Research Innovation and Impact (CARRii)

Established in February 2025, CARRii aims to shift the focus of UK respiratory research away from generating evidence in isolation, and towards embedding that evidence rapidly and equitably into clinical practice. Its creation reflects the realisation that research is not enough; implementation must become a priority if the NHS is to improve its capacity to cope with winter pressures.

**Fig. 1:**
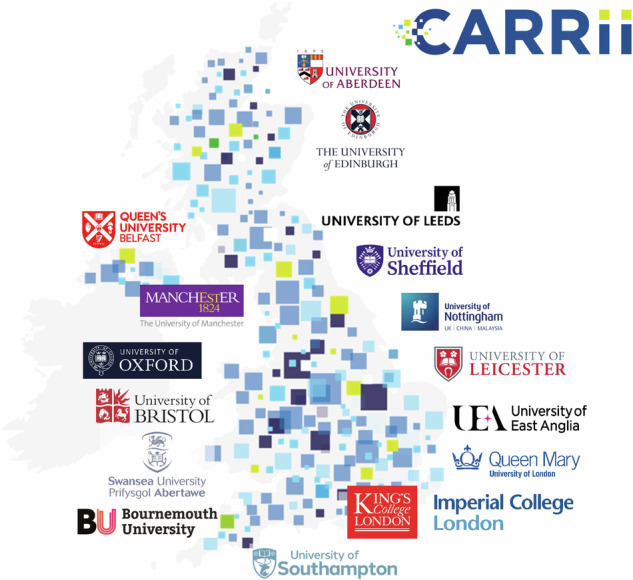
CARRii's UK-wide academic network. CARRii’s academic network.

CARRii is a UK-wide network bringing together leading university departments in respiratory research, clinical medicine, public health, data science and health economics (see Fig. 1), NHS and non-NHS organisations, industry innovators, and people living with respiratory conditions – in collaboration with the Association of British Health Industries and the charity Asthma and Lung UK. It is a ‘centre without walls’, based within the Nuffield Department of Primary Care Health Sciences at the University of Oxford. All members share a commitment to transforming respiratory care across the UK by driving policy change, prioritising implementation, designing interventions that work within routine practice, generating real-world evidence, and involving patients from the outset.

**CARRii’s key missions**

- To reduce NHS winter pressures and the burden of unscheduled care.
- To tackle respiratory inequalities.

**CARRii’s key themes**

- **Prevention**. Improvement in vaccination rates requires an understanding of what drives vaccine uptake^67,68^, improved engagement with communities, and high quality vaccination services^85^. Reducing respiratory virus transmission requires behavioural changes including hand washing, social distancing and the use of face masks^39,86^. Prevention also requires management of at-risk populations including younger children and older adults^20^, those living in inadequate housing and with health inequalities^20,41–45^, the housebound^31^, and people with immunosuppression and multimorbidity^20^. Community pharmacists have a significant role to play in medicines optimisation for those who are housebound and vulnerable^87^. In COPD, composite individual exacerbation risk scores such as ACCEPT^88^ and PRECISE-X^89^ can predict the risk of a first severe exacerbation^88^ and the rate and severity of exacerbations^89^, thus enabling proactive personalised COPD management aimed at preventing hospital admission. However, in asthma, a recent cluster randomised trial of a practice-based intervention for people at risk of hospitalisation or death showed no overall benefit, prompting further research and analysis^90^.
- **Self-management and connected care**. Despite good evidence of improved healthcare outcomes, implementation of supported self-management in asthma and COPD has been poor^49,50,72^. A systematic review of asthma self-management implementation studies^91^ and subsequent qualitative work^92^ showed that effective interventions combine active patient engagement with trained, motivated healthcare professionals working in a supportive environment with appropriate remuneration and protected time. The most effective model is regularly-supported self-management, which reduces healthcare resource use and improves quality of life across all levels of asthma severity^93^. In COPD, a recent Cochrane review concluded that self-management interventions are associated with improvements in health-related quality of life and a lower risk of hospital admission^94^. More work is needed to change perceptions and convince healthcare managers of the benefits of supported self-management^91–94^.
- **Optimising clinical care**. This is paramount. Improved clinical care all year round should mitigate the effects of winter virus infections^49,50^ and reduce winter pressures on the NHS. It requires system improvement at all levels, from national to local level and ultimately down to the patient consultation. Finland’s 10-year asthma and COPD programmes are the exemplar for nationwide system improvement^95,96^. For clinicians, it means practising evidence-based medicine and putting patients’ best interests first. Sacket *et al*. expressed it perfectly 30 years ago: “Evidence-based medicine is the conscientious, explicit, and judicious use of current best evidence in making decisions about the care of individual patients.”^97^ Provision of resources for regular clinical audit^72,73,77,78^, improved access to POCTs in primary care^69–71^, implementation and evaluation of new service models such as Acute Respiratory Infection hubs^83,84^, and implementation of pre-existing research evidence (such as exists for PR in COPD)^79–82^, are all essential. Newly published research which could achieve significant clinical impact needs to be identified quickly. For example, a recent large primary care RCT involving nearly 14,000 participants showed that using a gel-based or saline nasal spray during an acute respiratory illness reduces the duration of illness by nearly two days^98^ – a simple evidence-based intervention which CARRii is currently helping to disseminate and implement. Quality care should be effective, safe, and people-centred, and interventions should be timely, equitable, integrated, and cost-effective^99^. Clinical and cost-effectiveness studies are crucial if policymakers are to be convinced of the need for change. Two recent studies – one on the value of primary community health care nurses^100^, and another on a home-based health promotion intervention for older people with frailty^101^ – are good examples. The prerequisite for much of this change is a more innovative approach to data collection and an improvement in the quality of NHS IT systems which can often be overly time-consuming and burdensome^102^.

**CARRii’s specialist platforms**

CARRii incorporates an inter-disciplinary team of experts to ensure that its research methods and data infrastructure are of the highest quality:

- **Methodology**. Appropriate high-quality real-life research methods^60–64,69,97,98^ and cost-effectiveness evaluations^100,101^ are essential if research is to have academic credence, relevance to primary care and policy makers, and be implementable. Future work needs to include methods appropriate for environmental epidemiology, exposome immunology^103^, AI and risk prediction modelling^104^, and biomarker identification and implementation.
- **Data Science**. The power of data science is clear from the use of large datasets such as the EAVE II Scottish national linked dataset during the COVID-19 pandemic^32^.
- **Postgraduate Training**. A thriving applied clinical research community with training opportunities for early- and middle-career researchers brings increased research capacity, fresh perspectives, and helps train the next generation of respiratory scientists, clinical academics, and clinicians.
- **Patient and Public Involvement**. For research to be truly relevant to patients, patient and public involvement is essential from the outset^97,105,106^.

## Inaugural Annual Scientific Meeting (ASM), 29^th^ October 2025

Held at St Hilda’s College, Oxford, CARRii’s inaugural ASM brought together patient representatives, clinicians, researchers, policy and public-health experts, and industry partners. The discussions highlighted not only the scale of the preventable respiratory burden, but also the opportunity for practical action if research, innovation and policy can be aligned.

A consistent theme was the need to move beyond isolated pilots and into system-wide adoption. Presentations and panel discussions identified three persistent barriers. Firstly, real-world evidence is still limited. Clinical trials often exclude the complexity of everyday practice. Health services need to understand how interventions perform across different populations, settings and resource constraints. Secondly, implementation requires broad coalitions. Air-quality interventions depend on local government; housing improvements require multi-agency coordination; community-based care involves voluntary, social-care and primary care partners; and introducing new diagnostic tools requires commercial and regulatory alignment. No single institution can achieve impact alone. Thirdly, policy engagement is often too late. Evidence competes for limited political attention. Policy experts speaking at the meeting emphasised the need for concise, actionable insights and the importance of convincing policy makers that evidence-based improvements in clinical care align with government priorities, including the shift from analogue to digital care, from treatment to prevention, and from hospital-based to community-based models as per the NHS 10-year plan^107^.

Several research presentations underscored how respiratory health is inseparable from social and environmental determinants. Underheated homes are linked to increased infection rates in preschool children, and first reports from the CHILL study demonstrate the importance of clean-air policies for lung development in children and the wider societal benefits that could accrue^108^. Respiratory health is shaped by the conditions in which people live, learn and work. Addressing these inequalities requires action well beyond the NHS: investment in housing quality; urban planning that prioritises clean air; community-based vaccination programmes; and education that supports early symptom recognition and self-management. Respiratory research must therefore work across sectors if it is to reduce winter pressures.

## Challenges and priorities for the future

In the ASM’s closing session, participants were asked which interventions would most effectively reduce winter pressures in both the short- and long-term. Their priorities were striking in their pragmatism, and the subsequent discussion focussed on the improvements in clinical care which would result:

- improve vaccination uptake, especially among children
- strengthen public health communication on infection prevention
- expand clean-air initiatives
- support early identification of high-risk patients
- prioritise home- and community-based early care
- accelerate adoption of rapid point-of-care diagnostics
- create standardised, scalable ‘prevention bundles’
- enable integrated data across community, primary and secondary care

These solutions require alignment, coordination and a commitment to implementation. Health systems globally are grappling with how to translate evidence into action more effectively, especially for common and preventable conditions. The UK’s winter pressure crisis exposes systemic vulnerabilities, but it also offers an opportunity to show how multidisciplinary, implementation-focussed research networks such as CARRii can drive change.

## Conclusion

By positioning implementation and impact as a central scientific goal, CARRii aims to show how health systems can move from discovering respiratory solutions to delivering them at scale. If adopted widely and supported through policy, investment and cross-sector collaboration, this approach could reduce winter pressures, improve health equity and strengthen system resilience.

## Data Availability

No datasets were generated or analysed during the current study.
